# Self-partner inclusion predicts performance of romantically involved individuals in a body-scaled action-anticipation task

**DOI:** 10.1371/journal.pone.0251425

**Published:** 2021-05-18

**Authors:** Cédric A. Bouquet, Melissa Lafleur, Virginie Quintard, Stéphane Jouffre, Yannick Wamain, Yann Coello, Lucette Toussaint

**Affiliations:** 1 Université de Poitiers, Centre National de la Recherche Scientifique, Centre de Recherches sur la Cognition et l’Apprentissage UMR 7295, Poitiers, France; 2 Université de Lille, Centre National de la Recherche Scientifique, Sciences Cognitives et Affectives UMR 9193, Lille, France; University of Minnesota, UNITED STATES

## Abstract

Previous research has shown that romantic relationships can lead to the cognitive inclusion of a romantic partner into one’s own self-representation, resulting in blurred boundaries between self and intimate other. Recent work suggests that this self-other integration process encompasses the two dimensions of the self–the conceptual and the bodily self. In line with this, it has been proposed that romantic love is associated with cognitive states that blur or reduce the saliency of self-boundaries in the bodily domain. The present study tested this hypothesis by investigating the influence of the self-other integration process in romantic love on passability judgments of door-like apertures, an action-anticipation task that rests on the representation of bodily boundaries. Romantically involved and single participants estimated whether they could pass through apertures of different widths. Moreover, inclusion of romantic partner in the self was assessed using the Inclusion of Other in the Self (IOS) scale. The pattern of correlation and the ratio between participants’ shoulder width and aperture judgments did not differ between romantically involved participants and singles. However, our results revealed that in romantically involved participants, the relationship between individuals’ shoulder width and aperture judgements was moderated by IOS scores. A greater inclusion of romantic partner in the self was associated with a weaker prediction of aperture judgment by participants’ shoulder width. A similar moderating effect of the intensity of romantic feelings (as measured by the passionate love scale) on shoulder width-aperture judgment relationship was found. IOS scores, but not romantic feelings, also moderated aperture judgments made for another individual (third person perspective). Together, these findings are consistent with the view that inclusion of romantic partner in the self triggers cognitive states affecting self-boundaries in the bodily domain.

## Introduction

Romantic (passionate) love, defined as a “state of intense longing for union with another” [1, p.5], is a fundamental drive associated with specific behavioral and psychological traits that differentiate it from other types of love such as companionate love or maternal love [2–5]. Importantly, research indicates that romantic love powerfully affects individuals’ sense of self [6–8]. In the present study our goal was to investigate how the self-other integration process associated with romantic love influences who we are and more specifically how it affects the cognitive representation of the physical aspect of self-boundary.

Various models of romantic love have been proposed by social psychologists [e.g. 5,9]. Central to the current research is the “including the other in the self” approach to close relationships, which suggests that individuals incorporate aspects of their romantic partner into their own self-representation, creating overlapping cognitive structures of self and partner [7,10–12]. This approach first rests on the premise that individuals have a fundamental motivation to expand their sense of self in order to increase their potential efficacy, so that they seek opportunities to acquire new perspectives, resources, and identities [13,14]. Second, it is assumed that a major opportunity for self-expansion is provided by romantic relationships in which the self expands through the cognitive inclusion of the romantic partner in the self, such that the partner’s resources, perspectives, and identities, are to some extent experienced as one’s own [7,11,15,16]. By fulfilling the need to expand one’s self efficacy, the experience of such self-expansion through the inclusion of partner’s characteristics into the self would promote relationship satisfaction and as a rewarding, positive experience, it may also foster the exhilaration associated with romantic love [12,17,18]. In sum, romantic love would involve the successful inclusion of the other in the self [12].

Supporting the model of romantic love as an inclusion of the other in the self, there is ample evidence that in romantic lovers, the existing self-representation expands by incorporating a romantic partner’s characteristics, such that individuals’ description of who they are is enriched by the addition of previously unshared attributes of the romantic partner [19]. Critically, including partner’s characteristics into one’s self content means that the representational structure of the self shares elements–or overlaps–with the representational structure of the intimate other, which results in blurred distinction between self and romantic partner [13,20]. In line with this, studies have shown confusion between a partner’s and one’s own traits, interests, or attitudes [15,20,21; see also 22–27].

Another line of research based on the Inclusion of the Other in the Self (IOS) scale–a single-item pictorial measure commonly used to capture self-other integration [28]–confirms that romantically involved individuals demonstrate a prominent inclusion of the intimate other in the self, with participants reporting greater overlap with their romantic partner compared to with a close friend, siblings, or parents [29,30]. Importantly, IOS scores appear to be a predictor of relationship stability over 3 months [31], confirming the functional role of self-partner integration in romantic love.

There is thus evidence for intertwined representations of self and romantic partner with respect to individuals’ characteristics (attitudes, opinions, personality traits,…) that form what has been termed the conceptual or narrative self [32,33]. The conceptual self refers to an abstract, higher-level representation of the self associating information such as personality traits, beliefs, preferences and autobiographical memories [32,34–36]. However, current theories of selfhood argue for the existence of another dimension of self, referred to as the bodily self, which rests on body-related representations and sensorimotor processing [32,35,37–41].

Copious empirical and theoretical research suggests the existence of shared/overlapping representations of one’s own body and that of others [38,42–44]. A large body of work indeed shows that the neural networks which code for one’s own bodily states (actions, sensations, emotions) are also activated during the observation of someone else experiencing those states [42–44]. At the behavioral level, it is also well known that people tend to automatically imitate others’ bodily postures and states, which thus implies shared bodily states and corresponding motor representations between the mimicker and the one mimicked [45,46]. Various theoretical frameworks suggest that these overlapping body-related representations of self and other would provide a first-person experience of other’s states, and as such form the basis of understanding others and, more broadly, of social cognition processes [44,47]. Overlapping and confounded representations of one’s own and romantic partner’s bodily states is what is expected from including other in the self at an embodied, bodily level. This self-partner integration at the bodily level may have social-cognitive consequences that would be beneficial for the relationship, such as increased liking, trust, mutual understanding and cooperation [48].

There is accumulating evidence for an embodied self-other overlap in romantic love [for a review, see 48]. A first line of evidence comes from neuroimaging studies that have revealed common activations between the processing of one’s own and partner’s bodily states [49,50]. For instance, it has been found that imagining one’s romantic partner (vs. a stranger) in a painful situation was associated with greater involvement of the pain matrix activated when imagining one’s self in the same situation [49]. Another line of evidence comes from a well-known study in which it was revealed that, after twenty years of marriage, partnered individuals tend to become physically similar [51]. This finding may be accounted for by reciprocal imitation of the partner’s facial and other bodily expressions over time, leading romantic partners to incorporate the bodily expressions of the other in their own body representation. A particularly interesting hypothesis has been formulated by Burris and Rempel [52] regarding the consequences of including an intimate other in the self. Accordingly, including the romantic partner in the self “is associated with a greater sense of the self extending beyond the default limits defined by the perimeter of the physical body” [52, p. 947]. More specifically, it is assumed that there exists a psychological boundary that differentiates and protects the self, including the bodily self [52,53]. Consequently, the salience of the physical aspect of self-boundary should decrease in order to allow self-other inclusion in romantic relationships. In line with this proposal, the authors found that, as compared to singles, romantically involved individuals reported to feel their physical body as less constraining. Moreover, a negative correlation between the sense of physical vulnerability and body size (height and weight) was detected among singles, but not among partnered individuals [52]. Thus, these findings support the idea that due to the cognitive inclusion of the intimate other into the self, the representation of the bodily aspect of self-boundary gets less salient in romantic lovers [52]. Extending this view, behavioral studies have reported a blurring of the boundaries between self and romantic partner at a bodily level. For instance, romantically involved individuals have been found to demonstrate greater automatic imitation of their partner’s actions, as compared to a friend’s actions [54], suggesting a reduced ability to differentiate between the representations of one’s own and partner’s bodily states. Similarly, in the context of joint action, individuals take their romantic partner’s action into account to a greater extent than that of a close friend, confirming a reduced self-other distinction at the level of bodily self-representations in romantic love [30,55]. Romantic love may thus be associated with cognitive states that blur or reduce the saliency of self-boundaries in the bodily domain, in a way that is compatible with an inclusion of romantic partner in the self [48]. The aim of the present study was to further test this hypothesis by investigating whether perceptual judgments that rest on the representation of body’s spatial boundaries, such as decision about potential actions, are affected in romantically involved individuals. We reasoned that if the boundaries of the bodily-self get blurred or less salient, this should affect the ability to make body-scaled action-anticipation (in that it necessitates to rely on the representation of the body’s spatial properties).

To probe the ability to make body-scaled action-anticipation, we used the passability judgment paradigm [56]. In this paradigm, participants are asked to make perceptual judgments about the passability of door-like apertures (i.e. to decide whether or not the aperture is wide enough for them to pass through without turning their shoulders). Participants usually succeed in this task, choosing an aperture width allowing them to pass through it, with a safety margin. More precisely, it has been repeatedly found that these passability judgments derive from information about one’s own body, especially shoulder width, such that the perceived critical aperture/shoulder width ratio (referred to as π_p_) is around 1.2 in human adults [56–59]. Though the passability judgment paradigm has been developed initially to assess affordance judgments in the framework of direct perception [56,60,61], subsequent work in cognitive psychology has treated the paradigm more as a visuo-motor imagery task resting on body-related representations. The present work adopts such a cognitive approach to the passability judgment paradigm. Within this framework, passability estimates have been proposed as a proxy to investigate the internal representation of the body’s spatial properties [57–59,62,63]. Consistent with this proposal, passability judgements are adjusted to changes in body size that result for instance from carrying an object or large clothes [64,65].

Importantly, research further indicates that cognitive processes related to the spatial properties of the body can also be modulated by the social context and affective states [e.g. 58,66,67]. For instance, recent work has demonstrated that passability judgments in women were modulated by the degree of individuals’ bodily concerns and self-esteem, with greater concern and lower self-esteem being associated with a larger π_p_ [59,63]. In the same vein, it has been found that women suffering from anorexia demonstrate abnormal, increased π_p_, as compared to controls [57,62]. One can also consider another line of research on the representation of peripersonal space (the space around the body), which is tightly linked to the representation of the body’s spatial properties [66]. Research in this area indicates that the representation of peripersonal space varies according to personality traits, affective states and social context [68–72].

Together, these findings indicate that the processes that support passability judgments are not only constrained by the body’s metric properties, but are also sensitive to high-level social cognition and affective modulations. In the present study we thus used passability judgments of door-like apertures to behaviorally assess whether romantic love is associated with a blurring of the physical boundary of the body.

It is worth noting that we are not suggesting that such effects are constantly present in romantically involved individuals. It makes sense to assume that these effects, like other love-related phenomena, take place especially when interacting with the intimate other or, in the absence of the partner, when the romantic relationship schema and romantic feelings are activated, such as when individuals are reminded of their partner or their current relationship [73–75]. Following this reasoning, romantically involved participants from the present study were submitted to a priming procedure–writing a short essay–intended to activate thoughts for their partner and feelings of love [73,75,76]. A similar procedure directed to a friend was used for singles. Although it makes difficult to disentangle the effect of status (in-love vs. single) *per se* from the effect of priming, we opted for this procedure to maximize the likelihood of obtaining the expected effects in this first attempt to demonstrate an impact of romantic love on passability judgments.

As stressed above, judging whether a door-like aperture is wide enough to pass through should be affected by a blurring or reduced saliency of physical boundaries of the body. We see two ways it could impact performance on aperture judgments in romantically involved individuals as compared to singles. First, the relationship between body’s metrics and aperture judgments could be altered. In other words, the usual link between shoulder width and perceived critical aperture may be less pronounced in romantically involved participants. A variant of this prediction is that blurred bodily boundaries would make body-scaled action-anticipation more difficult, thereby leading to more cautious responses [77], which would mean a larger margin of safety (i.e. a larger perceived critical aperture/shoulder width ratio or π_p_) in the context of passability judgments [67,78].

Moreover, although inclusion of other in the self is a feature of close relationships, there are still substantial variations among partnered individuals [11]. Consequently, the same should also be true of the blurring or reduced saliency of bodily boundaries, as it is assumed to be related to the self-other inclusion process. Therefore, in partnered individuals, the degree of inclusion of romantic partner in the self should modulate the relationship between shoulder width and aperture judgments. We predicted that a stronger inclusion of romantic partner in the self would alter the relationship between participants’ shoulder width and perceived critical aperture. Moreover, given that self-partner integration has been linked to relationship satisfaction and passion [12,18,29], we also tested the moderating role of the intensity of romantic feelings, as measured by the Passionate Love Scale (PLS) [3].

To test these predictions, we had romantically involved and single individuals perform a passability judgment task in which they had to estimate whether an aperture was large enough to allow them to pass through (without turning their shoulder). In order to establish the specificity of the effects, i.e. that they relate to participants’ own body (first person perspective or 1-PP), we also included a passability judgment task in a third person perspective (3-PP), in which participants had to estimate whether the aperture was large enough to enable another individual (the experimenter) to pass through. Inclusion of partner in the self was evaluated through the IOS scale, a common and highly reliable tool to capture self-other inclusion [11,28,79]. Finally, because the passability judgement task requires to imagine an action, we sought to control for potential group difference in terms of motor imagery. To this end, we had participants complete the Vividness of Movement Imagery Questionnaire-2 (VMIQ-2), a psychometric tool assessing the ability to produce images of action [80]. The VMIQ-2 comprises two scales. The Internal visual imagery (IVI) scale assesses the ability to imagine one’s self performing an action (internal imagery). The External visual imagery (EVI) scale assesses the ability to imagine someone else performing an action.

## Materials and methods

### Participants

One hundred and fifty-three participants were recruited. We tested as many participants as possible during the semester, with a minimum requirement of 50 participants per group. Our final sample size involved more than 70 participants per group, limiting the likelihood of obtaining a false-positive result in the between-group comparison [81]. Moreover, with this sample size, we had a power greater than 0.85 to detect an interaction effect of medium size between Status and Shoulder width in the regression analysis [82].

All participants were students from the University of Poitiers, taking part in the experiment in exchange for course credit. Before starting the experiment, participants were asked whether they were currently involved in a romantic relationship, which was defined as “being in love” with someone and having an exclusive relationship with this person. Eighty-two participants were singles (M_age_ = 19.506, SD_age_ = 2.135; 41 women) and 71 were currently involved in a romantic relationship (M_age_ = 19.728, SD_age_ = 1.658; 41 women; mean length of romantic relationship = 23.41 months, SD = 18.18. Romantically involved participants declared themselves as heterosexual. On the 15-item version of the Passionate Love Scale (PLS) [3], romantically involved participants reported an average score of 108.35/135, SD = 12.97, which indicates that they were on average passionately in love with their romantic partner (score above 106 [83]).

Romantically involved participants did not differ significantly from singles in terms of shoulder width, *t*(151) = -1.025, *p* = .306 (Mean = 44.828 cm, SD = 3.367 vs. 44.230 cm, SD = 3.534, respectively). The IVI and EVI scores obtained in the VMIQ by romantically involved participants (mean IVI-score = 47.154, SD = 15.811; mean EVI-score = 44.225, SD = 14.842) were not significantly different from those measured in singles (mean IVI-score = 48.269, SD = 13.162; mean EVI-score = 44.925, SD = 12.276), both *t*s < 1, *p*s > .3. Thus, the two groups did not differ in terms of ability to imagine actions, as indexed by VMIQ scores. No further analysis was conducted on VMIQ scores.

Written informed consent was obtained from all individual participants included in the study. The study has been approved by the Ethics Committee for Research Involving Humans of the Universities of Tours and Poitiers (CER-TP, n°201905RGPD). All aspects of this study were performed in accordance with the ethical standards set out in the 1964 Declaration of Helsinki. Furthermore, the study was conducted in accordance with national norms and guidelines for the protection of human subjects.

### Material and procedure

Each experimental session was individual and lasted approximately 30 minutes. Upon arrival at the laboratory, the participant indicated whether he/she was currently involved in a romantic relationship or whether he/she was single.

The session began with an induction task [75]. Participants involved in a romantic relationship were asked to recall for 45 seconds the first times they met with their partner. They were then asked to write three sentences about these memories. Single participants followed the same instructions but referring to a friend of the opposite sex (they were asked to choose a friend of the opposite sex, with no further details about this relationship). Cross-sex friendship was used to match the induction condition of romantically involved participants who were heterosexual.

Then participants performed the first-person perspective (1-PP) passability judgment task [57,62]. This task consisted in imagining an action without carrying it out. A video-projector connected to a PC was placed on the floor at 4m from a white wall on which visual stimuli were projected. Visual stimuli consisted in door-like apertures. Two series of increasing apertures and two series of decreasing apertures were projected. Each series consisted in a succession of apertures varying in width from 30 cm to 78 cm, with a 2 cm increment (i.e. 25 different apertures). Participants alternated between increasing and decreasing series. Half of participants started with apertures increasing in width and the other half with decreasing apertures. Participants stood upright behind the video projector, their arms along their body, and at a distance of 4.00 m of the wall on which the aperture was projected.

For each projected aperture, participants were instructed to state “yes” if they thought they could pass through without rotating their shoulders and “no” if they could not. A series was terminated after two consecutive answers different from the previous ones (i.e., a transition from “yes” to two “no” consecutive replies in descending series, and vice-versa). For each series, a perceived critical aperture was computed as the mean of the two apertures width that received the last yes and first no judgment in decreasing series and the last no and first yes judgment in ascending series [60].

Then the perceived critical apertures obtained in the four series were averaged for each participant as a first dependent variable (mean perceived critical aperture). We computed a second dependent variable, the passability ratio (π_p_), for each participant by dividing the mean perceived critical aperture by the participant’s shoulder width [56]. By controlling for shoulder width, π_p_ reveals whether a participant overestimates/underestimates the width of the aperture required to pass through, with a π_p_ less than 1 indicating an underestimation and a π_p_ greater than 1 an overestimation.

After performing the 1-PP passability judgment task, participants performed the 3-PP passability judgment task. In this version of the task, a female experimenter (21 year old; height = 166 cm; weight = 54.5 kg; shoulder width = 39.9 cm) stood behind the video-projector, which position was the same as in the 1-PP task (i.e. at 4 m from the wall on which visual stimuli were projected). Participants were asked to stand on a mark on the floor, placed 1m meter behind and slightly to the left of the experimenter (so that participants were able to see the aperture stimuli). Consequently, participants were approximately 1 m further from the wall than in the 1-PP task. This variation was not a major issue as we were not interested in absolute performance difference between 1-PP and 3-PP task. Then, the stimuli and procedure used in the 3-PP passability judgment task were the same as in the 1-PP task, except that for each aperture, participants were required to estimate whether the experimenter could pass through without turning her shoulder. In the 3-PP task, π_p_ was calculated by dividing the mean perceived critical aperture by the experimenter’s shoulder width.

After completion of the two judgment tasks, participants’ shoulders width was measured by the experimenter in a standardized manner with a ruler, the participant standing with his/her back on a wall, and his/her arms along the body. Participants were then asked to report both their height and weight, before completing the VMIQ-2.

Finally, before completing the PLS, romantically involved participants completed the IOS scale in reference to their romantic partner. The scale consists in a set of seven pairs of increasingly overlapping circles, with one circle representing the self and the other circle representing the target person (for full description, see [11,79]). In the present study these circles were respectively labeled as “me” and “she/he”. Participants were asked to select the pair of circles that best describes their relationship with their partner. The score ranged from 1 to 7, the larger the score, the greater the inclusion of partner in the self. Then, they completed the 15-item version of the Passionate Love Scale [3,83]. No IOS measure was administered to single participants.

It is worth stressing that all participants performed the 1-PP passability judgment task before the 3-PP task. Starting with the 1-PP task was motivated by the assumption of potential carry-over effects between tasks. Because our predictions focused on the 1-PP passability judgment task, we chose to maximize the likelihood of obtaining the expected effect by avoiding potential contamination from prior performance of the 3-PP task. We are aware that, conversely, because of order effects, this comes at the cost of the interpretability of the results obtained in the 3-PP passability judgment task.

## Results

Three types of analyses were performed on the data from each perceptual judgment task (1-PP and 3-PP). First, we contrasted, using *t*-tests, π_p_ obtained in singles with that obtained in romantically involved participants. Second, a factorial regression analysis was used to test whether participants’ status modulated the extent to which their shoulder width predicted perceived critical aperture. Third, we conducted two factorial regression analyses in order to determine whether aperture judgement of romantically involved participants was influenced by (i) the degree of inclusion of romantic partner in the self, as indexed by IOS score and (ii) the intensity of romantic feelings, as indexed by PLS score.

Prior to the analysis of π_p_, we checked for the presence of outlier participants (defined as scoring above or below 3 SD from the corresponding group mean). No participant was identified as outlier according to this criteria. The contrast of π_p_ in the 1-PP task revealed no significant difference between single (Mean = 1.187; SD = 0.195) and romantically involved participants (Mean = 1.169; SD = 0.185), *t*(151) = 0.575, *p* = .565, *d* = 0.094. Similarly, π_p_ obtained in the 3-PP task did not differ significantly between single (Mean = 1.217; SD = 0.157) and romantically involved participants (Mean = 1.191; SD = 0.164), *t*(151) = 0.994, *p* = .321, *d* = 0.161.

A factorial regression analysis was conducted with the perceived critical aperture in the 1-PP task as the dependent variable, and with Status (coded -0.5 for singles, + 0.5 for romantically involved), mean-centered participant’s shoulder width (; i.e. the sample mean subtracted from each individual observation) and their interaction as predictors [84,85]. Prior to the analysis, we checked for the presence of outlier participants using the studentized residual technique (*t* greater than |2.00|). Four participants were identified as outliers and removed from the regression analysis. This analysis revealed that shoulder width significantly predicted perceived critical aperture, b = .453, β = .206, *t*(145) = 2.574, *p* = .011. The size of perceived critical aperture was positively related to shoulder width. The main effect of Status was not significant, b = -.749, β = .056, *t*(145) = -.630, *p* = .529. The interaction between both factors was not significant, b = .044, β = -.015, *t*(145) = 0.124., *p* = .902. The same analysis conducted on 3-PP data indicated no significant relationship between shoulder width and perceived critical aperture, b = .071, β = .039, *t*(145) = -.471, *p* = .638. The effect of status was not significant, b = -1.595, β = .129, *t*(145) = -1.573, *p* = .118. The interaction between both factors was not significant, b = -.070, β = -.019, *t*(145) = 0.234., *p* = .815.

**Fig 1:**
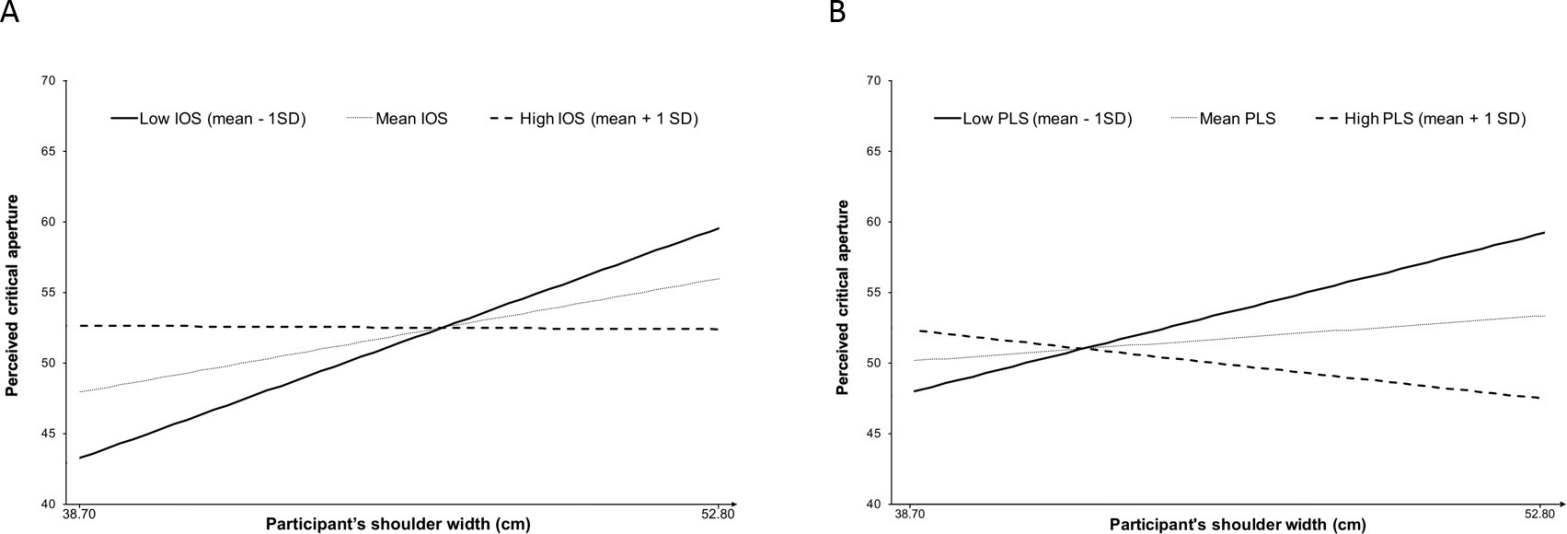
Moderating effects of IOS (A) and PLS (B) scores on the relationship between participants’ shoulder width and perceived critical aperture width in the 1-PP task. A: Shoulder width and IOS scores interactively predict perceived critical aperture in romantically involved participants. Participants’ shoulder width is a significant predictor of perceived critical aperture when IOS scores are small (mean– 1SD), b = 1.226, β = .550, but not when IOS scores are high (mean + 1SD), b = -.037, β = -.016. B: PLS scores also moderate the relationship between shoulder width and perceived critical aperture. This relationship is significant for low PLS scores (mean– 1SD), b = .846, β = .379, but not for high PLS scores (mean + 1SD), b = -.366, β = -.164.

A second factorial regression analysis was conducted on perceived critical aperture in the 1-PP task, with mean-centered participants’ shoulder width, mean-centered IOS scores and their interaction as predictors. One participant was identified as outlier for the regression analysis (studentized deleted residual *t* >|2.00|). The main effect of IOS was not significant, b = 1.040, β = .147, *t*(66) = 1.271, *p* = .208. The participants’ shoulder width was a significant predictor of perceived critical aperture, b = .595, β = .267, *t*(66) = 2.285, *p* = .026, but as expected, this effect was qualified by a significant interaction with IOS, b = -.591, β = -.250, *t*(66) = -2.159, *p* = .034. We thus examined the simple effect of shoulder width for two levels of IOS scores. When IOS scores are small (mean– 1SD), shoulder width is a significant predictor of perceived critical aperture, b = 1.226, β = .550, *t*(66) = 2.953, *p* = .004. Thus, when participants show a weak inclusion of their partner in the self, the wider the participant’s shoulder width, the wider the size of perceived critical aperture. In contrast, this positive relationship between participant’s shoulder width and critical aperture is not significant when IOS scores are high (mean + 1SD), b = -.037, β = -.016, *t*(66) = -.100, *p* = .921. Thus when participants show a high self-partner inclusion, their shoulder width does not predict significantly the size of perceived critical aperture (Fig 1A). We conducted a similar factorial regression analysis that included mean-centered PLS scores, instead of mean-centered IOS scores, as factor. This analysis revealed that the main effect of PLS scores was not significant, b = -.096, β = -.166, *t*(66) = -1.430, *p* = .157. The main effect of shoulder width was not significant, b = .240, β = .107, *t*(66) = .895, *p* = .374, but it was qualified by a significant interaction with PLS scores, b = -.046, β = -.279, *t*(66) = -2.369, *p* = .021. The relationship between shoulder width and perceived critical aperture is positive and significant for low PLS score, b = .846, β = .379, *t*(66) = 2.646, *p* = .010, while this relationship is non-significant when PLS score is high, b = -.366, β = -.164, *t*(66) = -.881, *p* = .381 (Fig 1B).

We repeated these analyses on the 3-PP data obtained by romantically involved participants. The analysis including IOS scores and participants’ shoulder width as predictors indicated that the main effect of shoulder width was not significant, b = .143, β = .078, *t*(66) = .658, *p* = .513, nor the main effect of IOS score, b = .984, β = .170, *t*(66) = 1.442, *p* = .154. However, the interaction between these two factors was significant, b = -.515, β = -.266, *t*(66) = -2.254, *p* = .027. When IOS scores are small, shoulder width is a significant predictor of perceived critical aperture, b = .693, β = .379, *t*(66) = 2.000, *p* = .050. Thus, when participants show a weak inclusion of their partner in the self, the relationship between participants’ shoulder width and perceived critical aperture is positive and significant. In contrast, when IOS scores are high, this relationship between shoulder width and critical aperture is not significant, b = -.401, β = -.222, *t*(66) = -1.333, *p* = .187. The same analysis conducted with mean-centered PLS scores, instead of mean-centered IOS scores, revealed no significant effect of participants’ shoulder width, b = -.106, β = -.058, *t*(66) = -.460, *p* = .647, and no significant effect of PLS, b = -.045, β = -.096, *t*(66) = -.785, *p* = .435. These two factors did not interact significantly in the prediction of perceived critical aperture, b = -.031, β = -.230, *t*(66) = -1.820, *p* = .073.

Complementary analyses were conducted to test for the potential influence of gender, age and relationship length on the above-described interactions between shoulder width and IOS/PLS. Because we had no specific predictions regarding how these factors may interact with our main variables (shoulder width, IOS, PLS), we conducted hierarchical factorial regressions as follows. Our critical predictors (shoulder width and IOS or PLS, and their interaction) were entered in the regression at step 1. Then, the factor Gender, or Age, or Relationship length, and its interactions with our critical predictors were entered at step 2 in order to test whether including this factor in the regression explained a statistically significant amount of variance in perceived critical aperture. These analyses revealed that the interaction between IOS and shoulder width in both the 1-PP task and in the 3-PP task was not modulated by Age (*p*s > .543), nor by Gender (*p*s > .660), nor by Relationship length (*p*s > .657). The interaction between PLS and shoulder width in both the 1-PP task and in the 3-PP task was not modulated by Age (*p*s > .179), nor by Gender (*p*s > .316), nor by Relationship length (*p*s > .791).

## Discussion

There is accumulating evidence that romantic love blurs self-partner boundaries in the bodily domain [30,48,54]. Completing this view, it has been proposed that romantically involved individuals show a reduced saliency of self-boundaries in the bodily domain, in a way that is compatible with self-other integration [52]. We further tested this hypothesis using passability judgments of door-like apertures [56]. We reasoned that if the boundaries of the bodily-self get blurred or less salient, this should affect the ability to make passability judgments of door-like apertures, since this kind of action-anticipation task necessitates to rely on the representation of the body’s boundaries [57,61–63].

A first prediction was that the classical link between participant’s shoulder width and perceived critical aperture would be attenuated in romantically involved participants, as compared to singles. A related prediction was that blurred or less salient bodily boundaries would lead romantically involved participants to adopt a larger margin of safety (larger π_p_) than singles in passability judgments [67,78]. Our results did not confirm these predictions. Indeed, we found no significant effect of participants’ status (single vs. romantically involved) on the link between participant’s shoulder width and perceived critical aperture (first prediction) nor on π_p_ (second prediction).

We further hypothesized that in romantically involved individuals, the blurring/reduced saliency of self-boundaries should critically depends on the extent to which romantic partner is included into the self. In other words, the altered shoulder width–critical aperture relationship, which we considered to be indicative of altered self-boundaries, would be prominent in individuals showing a strong inclusion of the intimate other in the self. In line with this hypothesis, the analyses of performance of romantically involved participants in the 1-PP task revealed that the strength of the link between participants’ shoulder width and perceived critical aperture was negatively affected by the amount of inclusion of romantic partner in the self, as indexed by IOS scores. Converging evidence was obtained when testing the modulatory role of PLS scores. We found that the relationship between participants’ shoulder width and perceived critical aperture in the 1-PP task was negatively affected by the intensity of romantic feelings (as indexed by PLS score).

The fact that IOS and PLS scores had a similar modulatory effect on 1-PP data is consistent with the relationship that has been established between inclusion of romantic partner in the self and romantic feelings [12,18,29]. Furthermore, the influence of the intensity of romantic feelings, as indexed by PLS, on perceptual judgments is in line with recent work suggesting that the positive affective states associated with romantic love may affect cognitive functioning [48].

In the light of this modulatory effect of IOS and PLS scores, it is not surprising that we did not find differential patterns of correlation as a function of group (singles vs. involved) nor differences in terms of π_p_. Indeed, it suggests that only those romantically involved individuals showing a strong inclusion of romantic partner in the self or strong romantic feelings would differ from singles in the extent to which their body’s metrics predict passability judgments.

The pattern of correlation between IOS scores, shoulder width and passability judgments in the 1-PP task is thus compatible with the idea that including one’s partner into the self triggers mechanisms affecting self-boundaries in the bodily domain [30,48,52]. Moreover, it is worth noting that inclusion of other in the self was indexed by IOS score, which taps conceptual forms of self-representation [79], while passability judgments were used to probe representations related to the bodily-self. Hence, consistent with previous work, our results suggest an interaction between conceptual- and bodily-self [35,86,87].

Additionally, in the 3-PP task, we had participants infer the passability of another person, in order to assess their accuracy in making passability judgments that did not relate to their own body [62,63]. We found that, for the studied population as a whole, participants’ shoulder width was not a significant predictor of perceived critical aperture in the 3-PP task, confirming that, overall, participants did not use information about their own body size to perform this task (or at least to a lesser extent than in the 1-PP task). From there, one may expect that if the moderation of romantically involved participants’ 1-PP task performance by PLS and IOS scores was related specifically to participants’ own body, these two factors should have no effect on 3-PP data. Consistent with this prediction, we found that PLS scores did not significantly moderate the relationship between participant’s shoulder width and critical aperture in the 3-PP task. In contrast, IOS scores had a significant moderating effect in this task, such that the relationship between shoulder-width and critical aperture was not significant as IOS scores increased, while the relationship was positive and significant as IOS scores decreased. To the extent that for high levels of IOS, participants’ shoulder width was not a significant predictor of aperture judgments in the 3-PP task, one may infer that in this task, participants with strong inclusion of romantic partner in the self related–as required–to information about the other’s body instead of information about their own body. In turn, this implies that in the 1-PP task, the absence of a significant shoulder width-aperture judgment relationship found for high IOS scores cannot be attributed to a generic process affecting the processing of bodies or body-scaled action anticipation in general. More intriguing is the finding that in the 3-PP task, for lower levels of IOS, participants’ shoulder width was significantly and positively related to passability judgments. A hint to a possible explanation can be found in previous empirical and theoretical research that has identified categories of individuals who are more self-centered and less prone to self-other integration (see for instance the distinction between inclusive vs. non-inclusive identities [88] or between individualistic vs. interdependent self-construal [89]. Hence, it is possible that a weak inclusion of romantic partner into the self revealed a specific psychological profile characterized by a tendency to be more self-centered (and more prone to maintain a distinction between self and others). Such a self-centered bias in individuals low in IOS may lead them to be anchored in their self-perspective and rely on their own body when making passability judgments in the 3-PP task. This explanation is however speculative and warrants further investigation. Finally, it is important to remember that the 1-PP task was always performed before the 3-PP task. Thus passability judgments in the 3-PP task were possibly influenced by prior judgments in the 1-PP task, which calls for caution in interpreting the 3-PP data.

An important limitation of the present study is that single participants were not administered an IOS measure in reference to their friend. This prevents us from testing whether the self-other inclusion process in the context of friendship [20,79] would have the same moderating impact as self-other inclusion in romantic couples on the link between participants’ body metric and aperture judgments. Thus, we cannot conclude on whether the present findings depend on the self-other inclusion process in romantic relationships or in close relationships in general.

However, it should be noted that romantic love is associated with specific behavioral and psychological traits, such as an intense focusing on the partner, intense emotions, and sexual desire, distinguishing it from other forms of close relationship [1–5]. Moreover, partnered individuals show greater cognitive overlap with their romantic partner compared to with a close friend, siblings, or parents [29,30]. Thus, the effects of self-other inclusion in friendship may be quantitatively and/or qualitatively different from those associated with the inclusion of one’s romantic partner in the self.

Furthermore, due to the correlational nature of our main finding, we can only speculate about the causal link between inclusion of romantic partner in the self and blurred bodily boundaries. One possibility is that including one’s partner into the self is associated with cognitive mechanisms that affect different dimensions of self-representation. As suggested by Burris and Rempel [52], the physical body plays a crucial role in psychological boundaries between self and not-self. They further suggest that the self-expansion resulting from the cognitive inclusion of an intimate other in the self challenges the boundary of the self, which leads romantically involved individuals to be less focused on themselves as separate entities and show a reduced saliency of bodily boundaries. Such an effect on bodily boundaries may vary as a function of the degree of self-partner inclusion, accounting for the pattern of correlations we observed in romantically involved participants. Alternatively, it is also possible that participants who demonstrate blurred or less salient self-boundaries are more prone to include their intimate partner in the self. In order to demonstrate the effect of the inclusion of romantic partner in the self on perceptual judgement of aperture passability, future research could for instance focus on romantically involved participants and use a priming procedure aiming to strengthen or reduce the distinctness between self and partner (see for instance [52] Experiment 5) before performance of the action-anticipation task.

Another interesting line of future research is to consider the possibility of an inclusion of romantic partner in the self at a bodily level such that, paralleling what has been documented at the level of the conceptual self (see introduction), romantic lovers would tend to incorporate some of the partner’s bodily features into their own self-content. In line with this proposal, recent work has demonstrated that the cognitive overlap between self and close others extends to the representation of faces [90]. Hence, an intriguing question is whether the degree of physical dissimilarity between self and partner–implying more or less changes in self-content–is associated with differential effects on self-boundaries. One way to investigate this question would be to examine passability judgements in romantically involved participants, while taking into account similarity with the partner’s body metrics. It would be particularly relevant to examine whether self-partner difference in terms of shoulder width contributes significantly to the prediction of passability judgments.

Finally, given that no significant difference was found between single and romantically involved individuals, and due to the limitations described above, we consider the current findings as preliminary and calling for further research.

In conclusion, the present work adds to a growing body of work suggesting that the process of self-other inclusion in romantic love involves cognitive changes that encompass the two dimensions of the self–the bodily and the conceptual self. Previous work investigating bodily-self in romantic love mostly focused on self-other distinction. In the present study, we focused on self-boundaries and investigated for the first time performance on a body-scaled action-anticipation task in the context of romantic love. While no difference with singles were revealed, we found that in romantically involved participants, passability judgments were predicted by an interaction between shoulder width and the level of self-partner inclusion. Our findings suggest that the effects of interpersonal closeness on self boundaries in the bodily domain are subtle and seem to depend on the degree of inclusion of the significant other in the self.
